# Spin-orbit interactions of transverse sound

**DOI:** 10.1038/s41467-021-26375-9

**Published:** 2021-10-21

**Authors:** Shubo Wang, Guanqing Zhang, Xulong Wang, Qing Tong, Jensen Li, Guancong Ma

**Affiliations:** 1 Department of Physics, City University of Hong Kong, Tat Chee Avenue, Kowloon, Hong Kong China; 2 Department of Physics, Hong Kong Baptist University, Kowloon Tong, Hong Kong China; 3 Department of Physics, The Hong Kong University of Science and Technology, Clear Water Bay, Hong Kong China

**Keywords:** Metamaterials, Acoustics

## Abstract

Spin-orbit interactions (SOIs) endow light with intriguing properties and applications such as photonic spin-Hall effects and spin-dependent vortex generations. However, it is counterintuitive that SOIs can exist for sound, which is a longitudinal wave that carries no intrinsic spin. Here, we theoretically and experimentally demonstrate that airborne sound can possess artificial transversality in an acoustic micropolar metamaterial and thus carry both spin and orbital angular momentum. This enables the realization of acoustic SOIs with rich phenomena beyond those in conventional acoustic systems. We demonstrate that acoustic activity of the metamaterial can induce coupling between the spin and linear crystal momentum **k**, which leads to negative refraction of the transverse sound. In addition, we show that the scattering of the transverse sound by a dipole particle can generate spin-dependent acoustic vortices via the geometric phase effect. The acoustic SOIs can provide new perspectives and functionalities for sound manipulations beyond the conventional scalar degree of freedom and may open an avenue to the development of spin-orbit acoustics.

## Abstract (web summary)

Spin-orbit acoustics is determinant to provide new perspectives and functionalities for sound manipulations. Here the authors theoretically and experimentally demonstrate acoustic spin-orbit interaction enabling chiral sound-matter interactions with unprecedented applications.

## Introduction

Spin and orbital angular momentum (OAM) are intrinsic properties of classical waves. Spin is associated with circular polarization (vector degrees of freedom) of waves and is characterized by the local rotation of a vector field. OAM originates from the spatial phase gradient (scalar degree of freedom) of waves and manifests as a helical wave front^1^. The couplings between spin and OAM, referred to as spin–orbit interactions (SOIs), can give rise to intriguing phenomena and applications in optics^2–8^, such as photonic spin-Hall effect^9–11^ and spin-dependent vortex generation^12,13^. SOIs are unique to transverse waves such as light and are absent for longitudinal waves. This is because although longitudinal waves such as airborne sound can carry OAM^14–18^, they are spin-0 in nature. Recent studies show that an engineered sound field can possess a locally rotational velocity field **v** that may be regarded as acoustic spin^19–21^, similar to electric spin deriving from the local rotation of electric field. Such an acoustic spin can emerge locally in nonuniform acoustic fields^20,21^ and has recently been observed in experiments^19,22^. In a homogenous medium, however, the spatial integration of acoustic spin density for a localized wave must vanish, in agreement with its spin-0 nature^20^. Despite this discovery of acoustic spin, SOIs remain beyond reach in sound, a fact that mainly owes to the lack of degrees of freedom. In other words, sound is characterized by a scalar pressure field *p* and a vector velocity field **v**, whereas light is characterized by two vector fields **E** and **H**.

In this work, we show that airborne sound can behave as a transverse wave with well-defined polarization in an acoustic metamaterial that goes beyond the Cauchy elasticity and follows a micropolar elasticity theory^23^. Unlike previous spin-sustaining acoustic fields^19,20,22^, the transverse sound is spin-1 in nature and carries the properties of elastic waves. It is characterized by two types of vector-field degrees of freedom, i.e., a velocity field and a microrotation field. The acoustic activity of the metamaterial can induce coupling between the velocity and microrotation fields, which can be considered an analog of chirality in electromagnetism (i.e., optical activity). Such a material property has recently been realized in elastic wave systems^24–27^ but is so far missing in acoustic wave systems. We theoretically and experimentally demonstrate two types of acoustic SOIs in momentum space and in real space, respectively. In the momentum space, the acoustic activity induces the coupling between spin and linear crystal momentum **k**, and enables the chirality-induced negative refraction, which was previously possible only in optical metamaterials^28,29^. In the real space, scattering of the circularly polarized transverse sound by a dipole particle can generate a sound vortex with a topological charge determined by the acoustic spin.

## Results

### Transverse sound

**Fig. 1:**
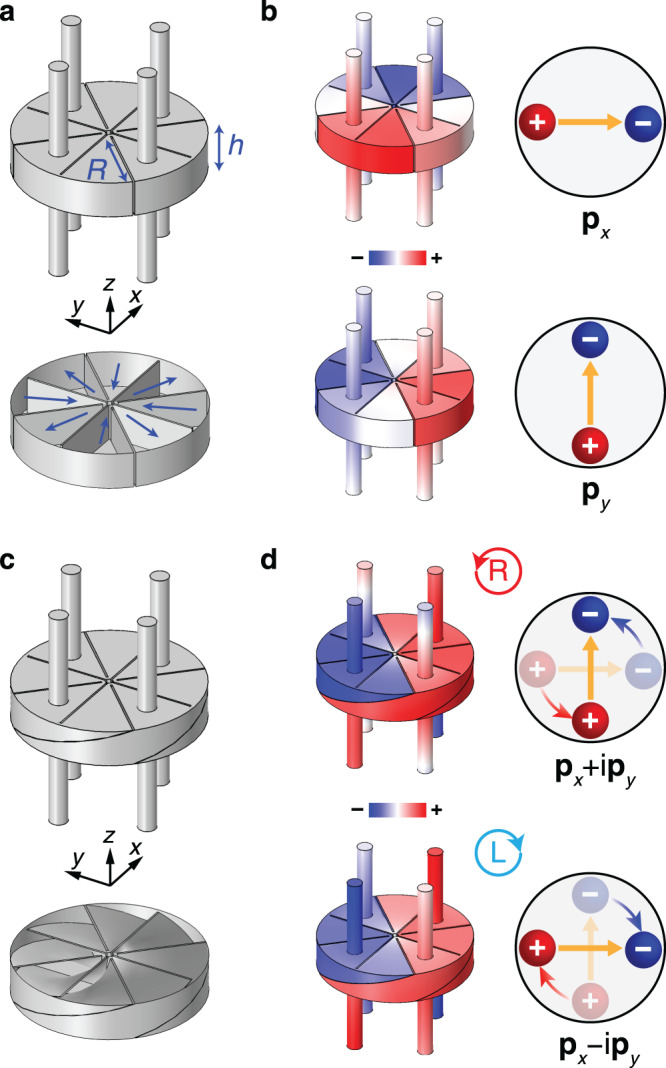
Eigenmodes of the 1D acoustic lattices. **a** The unit cell of the achiral lattice. The arrows show the flow of air inside the resonator. **b** The pressure eigenfields of the two transverse dipole modes. The velocity is linearly polarized on the transverse plane, corresponding to acoustic dipoles ${{{{{{\bf{p}}}}}}}_{x}$ and ${{{{{{\bf{p}}}}}}}_{y}$. The positive (negative) charge corresponds to positive (negative) pressure. The yellow arrows denote the velocity field. **c** The unit cell of the chiral lattice. **d** Pressure eigenfields of the chiral dipole modes. The velocity fields are circularly polarized on the transverse plane, corresponding to circularly polarized dipoles ${{{{{{\bf{p}}}}}}}_{x}\pm i{{{{{{\bf{p}}}}}}}_{y}$.

The longitudinal nature of airborne sound (${{{{{\boldsymbol{\nabla }}}}}}\times {{{{{\bf{v}}}}}}=0$) dictates that the velocity field **v** aligns with the direction of wave vector **k** in general. However, this is not necessarily true when sound is confined in a closed space. Consider a one-dimensional (1D) lattice stacked along the *z* axis with a unit cell shown in Fig. 1a. The unit cell consists of a cylindrical resonator with eight internal blades segmenting the air to achieve subwavelength resonance, as indicated by the blue arrows. The resonators are sequentially connected by four tubes. All solid–air interfaces are regarded as sound-hard boundaries. The resonator supports two degenerate and orthogonal dipole resonances with pressure eigenfields shown in Fig. 1b. The positive and negative pressure (indicated by the red and blue colors, respectively) induces an in-plane velocity field that is perpendicular to the propagating direction of sound (i.e., *z* axis). This corresponds to the oscillating dipole moments ${{{{{{\bf{p}}}}}}}_{x}$ and ${{{{{{\bf{p}}}}}}}_{y}$, where the positive (negative) charge corresponds to the positive (negative) pressure and the yellow arrow denotes the velocity field. Next, we break the spatial inversion symmetry by twisting the resonator geometry with respect to *z* axis, as shown in Fig. 1c. The degeneracy of ${{{{{{\bf{p}}}}}}}_{x}$ and ${{{{{{\bf{p}}}}}}}_{y}$ is removed, and the resonator supports two chiral eigenmodes ${{{{{{\bf{p}}}}}}}_{x}-i{{{{{{\bf{p}}}}}}}_{y}$ and ${{{{{{\bf{p}}}}}}}_{x}+i{{{{{{\bf{p}}}}}}}_{y}$, corresponding to a left-handed circularly polarized (LCP) dipole and a right-handed circularly polarized (RCP) dipole, respectively, as shown in Fig. 1d. Thus, the collective excitations of the acoustic dipoles in Fig. 1b, d will give rise to linearly polarized and circularly polarized transverse sounds propagating in the *z* direction, respectively.

**Fig. 2:**
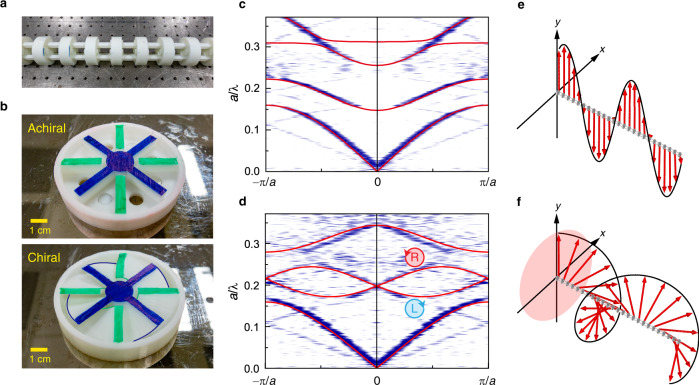
Band properties of the 1D acoustic lattices. **a** The fabricated sample of the 1D lattice. **b** The internal structures of the achiral and chiral unit cells. Experimental (blue) and numerical (red lines) results for the band structure of the achiral lattice (**c**) and the chiral lattice (**d**). The averaged velocity field of the achiral lattice shows a linear polarization (**e**), whereas circular polarization is seen for the chiral lattice (**f**).

To verify this, we use three-dimensional (3D) printing to fabricate both the 1D achiral and chiral lattices, each with 24 unit cells, as shown in Fig. 2a. In Fig. 2b, we show the cutaway views of the two types of unit cell, where the internal blades are colored to clearly show their orientations. The green-colored blades are connected to the outer shell and the blue-colored blades are connected to the inner core. They together form a tunnel in which air flows. The experimentally measured band structures of the achiral and chiral lattices are shown in Fig. 2c, d, respectively. The solid red lines denote full-wave numerical results calculated using a finite-element package COMSOL (see “Methods”). Excellent agreement between the experimental and numerical results is seen. The first band that extends to the static limit corresponds to a monopole mode, which has almost identical characteristics for both the chiral and achiral lattices. The second and third bands are the aforementioned transverse dipole modes, which are degenerate for the achiral lattice (Fig. 2c) but split into two bands for the chiral lattice (Fig. 2d) due to inversion symmetry breaking. The modes of the second and third bands for the chiral lattice are LCP and RCP, respectively. To obtain intuitive pictures of the transverse modes, we calculated the averaged velocity (near ${k}_{z}=0$) in each unit cell and plot it in Fig. 2e, f. Figure 2e shows the velocity field for the achiral lattice with 25 units, where the dipole mode along the *y* axis is excited. As seen, the sound is linearly polarized along the *y* direction with a wavelength much larger than the unit-cell dimension. We note that the achiral lattice also supports circularly polarized sound, which corresponds to a superposition of the linearly polarized sounds along the *x* and *y* directions. Figure 2f shows the velocity field for the second band of the chiral lattice, which clearly represents an LCP transverse sound. These confirm the transverse nature of the sound in the 1D lattices.

### Micropolar metamaterial with acoustic activity

**Fig. 3:**
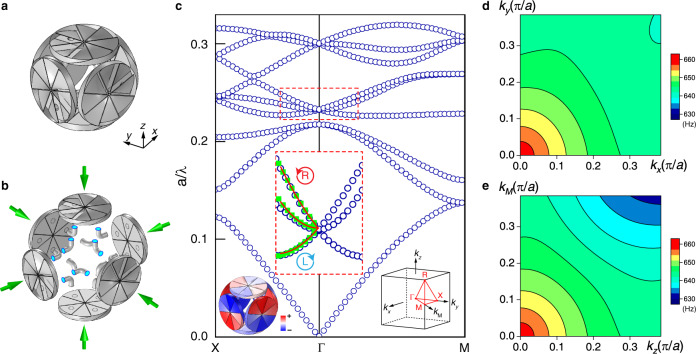
The 3D acoustic micropolar metamaterials. **a** The unit cell consists of three orthogonally arranged resonators connected with tubes. **b** The details of the unit-cell components. **c** The band structure of the metamaterial. The middle inset (dashed red box) shows a zoom-in of the dipole bands. The blue circles denote the numerical results of the metamaterial. The green squares denote the numerical results of the micropolar effective medium. The solid red lines denote the analytical results. The inset at the left corner shows the pressure eigenfield of the LCP mode. The inset at the right corner shows the Brillouin zone. **d** The isofrequency contours of the negative band in *k*_*x*_–*k*_*y*_ plane. **e** The isofrequency contours of the negative band in *k*_*z*_–*k*_*M*_ plane.

The above physics can be extended to the 3D metamaterial with the unit cell shown in Fig. 3a. The unit cell consists of three chiral resonators mutually connected with tubes, as shown in Fig. 3b. The numerically calculated band structure of the metamaterial is shown in Fig. 3c. The lowest three bands derive from the monopole mode of the chiral resonators (see Supplementary Information). The three bands enclosed by the red rectangle derive from the dipole modes. The upper and lower bands correspond to the RCP and LCP transverse modes, respectively, and the middle band corresponds to a longitudinal mode. The inset at the left corner of Fig. 3c shows the pressure eigenfield of the LCP mode at a time. In Fig. 3d, e, we plot the isofrequency contours of the LCP band in *k*_*x*_–*k*_*y*_ and *k*_*z*_–*k*_M_ planes, respectively. The contours are approximately circles for $k \, < \, 0.15\pi /a$, which indicates that the mode is isotropic near the $\Gamma$ point. The isotropic dispersions of the transverse modes are protected by time-reversal symmetry and chiral cubic symmetry^30^. At the frequencies of the transverse modes, the unit cell is subwavelength ($\sim0.23\lambda$). Thus, the metamaterial is macroscopically isotropic and homogeneous, and its material properties can be described by an effective medium theory. Remarkably, the emergence of wave transversality in the metamaterial implies the existence of a non-zero shear modulus for the effective medium, which is counterintuitive, as air does not generate shear forces. Here, the striking properties of non-vanishing shear modulus are induced by the transverse motion of sound enforced by the resonators with twisted internal blades. The existence of a non-zero shear modulus indicates that the metamaterial is equivalent to an elastic medium and the airborne sound behaves like an elastic wave with well-defined spin^31^. Because of its microscopic twisting feature, the metamaterial cannot be described by conventional effective medium theory based on Cauchy elasticity, which assumes symmetric stress and strain. Instead, micropolar elasticity (i.e., Cosserat elasticity)^23^, which is a high-order extension of Cauchy elasticity, can be employed to accurately characterize its unusual properties.

The micropolar elasticity assigns three rotational degrees of freedom to each material point in addition to the three linear degrees of freedom associated with displacement^23,32–34^. Each point is thus characterized by a displacement vector field $\,{{{{{\bf{u}}}}}}$ and a microrotation vector field ${{{{{\mathbf{\phi }}}}}}$. Using Einstein summation convention, the deformation of the medium can be expressed as:$\,{\varepsilon }_{{ij}}=\partial {u}_{j}/\partial {x}_{i}- {\epsilon }_{{ijk}}{\phi }_{k};{\kappa }_{{ij}}=\partial {\phi }_{j}/\partial {x}_{i}$, where ${\varepsilon }_{{ij}}$ is the asymmetric strain tensor, ${\kappa }_{{ij}}$ is the curvature tensor characterizing the relative microrotation between neighboring points, ${\epsilon }_{{ij}k}$ is the Levi-Civita symbol, and *i*, *j*, *k* iterate the Cartesian coordinates. For our micropolar metamaterial, the corresponding effective medium is characterized by the constitutive relations: ${\sigma }_{{ij}}={C}_{{ijkl}}{\varepsilon }_{{kl}}+{B}_{{ijkl}}{\kappa }_{{kl}},{m}_{{ij}}={B}_{{klij}}{\varepsilon }_{{kl}}+{D}_{{ijkl}}{\kappa }_{{kl}},$ where ${\sigma }_{{ij}}$ and ${m}_{{ij}}$ are the asymmetric force stress tensor and couple stress tensor, respectively^26,35^. ${B}_{{ijkl}},{C}_{{ijkl}}$ and ${D}_{{ijkl}}$ are the elastic constitutive tensors of the form ${X}_{{ijkl}}={X}_{1}{\delta }_{{ij}}{\delta }_{{kl}}+{X}_{2}{\delta }_{{ik}}{\delta }_{{jl}}+{X}_{3}{\delta }_{{il}}{\delta }_{{jk}}$ with $X=B,C,{D}$, and ${\delta }_{{ij}}$ being the Kronecker delta. Notably, ${B}_{{ijkl}}$ is a pseudo-tensor that characterizes the chirality of the medium and it changes sign under spatial inversion. Thus, the micropolar metamaterial possesses chirality that corresponds to the acoustic counterpart of optical activity^36^. Such a property has recently been realized in elastic metamaterials^24–26^ but has no acoustic counterpart to date. It is different from the Willis-type bianisotropy, in which the stress–strain couples with momentum–velocity^37–42^.

The propagation of the transverse sound is governed by the conservation of linear and angular momenta: $\partial {\sigma }_{{ji}}/\partial {x}_{j}=\rho {\partial }^{2}{u}_{i}/\partial {t}^{2}{{{\rm{;}}}}\,\partial {m}_{{ji}}/\partial {x}_{j}+{\epsilon }_{{ijk}}{\sigma }_{{jk}}{{{\mathscr{=}}}}{{{\mathscr{j}}}}{\partial }^{2}{\phi }_{i}/\partial {t}^{2}$, where $\rho$ is the mass density and ${{{\mathscr{j}}}}$ is the microinertia density (i.e., micro moment of inertia per unit volume). Assuming the time-harmonic displacement eigenfield ${u}_{i}={U}_{i}{e}^{{{{\rm{i}}}}{k}_{i}{x}_{i}-{{{\rm{i}}}}\omega t}$ and microrotation eigenfield ${\phi }_{i}={\Phi }_{i}{e}^{{{{\rm{i}}}}{k}_{i}{x}_{i}-{{{\rm{i}}}}\omega t}$, the dispersion relations of the dipole modes near the $\Gamma$ point (retained the lowest order of $k$) can be obtained as (see “Methods”): ${\omega }_{T}^{\pm }={\omega }_{0}\pm {vk}$ and ${\omega }_{L}={\omega }_{0}+\tau {k}^{2},$ where ${\omega }_{0}={\scriptstyle\sqrt{2\left({C}_{2}-{C}_{3}\right){{{\mathscr{/}}}}{{{\mathscr{j}}}}},{k}=\left|{{{\bf{k}}}}\right|={{{\bf{k}}}}/\hat{{{{\bf{k}}}}},v=\left({B}_{2}-{B}_{3}\right)/\sqrt{2{{{\mathscr{j}}}}\left({C}_{2}-{C}_{3}\right)},\tau =\left({D}_{1}+{D}_{2}+{D}_{3}\right)/}\scriptstyle\sqrt{8{{{\mathscr{j}}}}\left({C}_{2}-{C}_{3}\right)}$, and the subscripts “T” and “L” denote the transverse and longitudinal modes, respectively. It is seen that microrotation significantly impacts both the transverse and longitudinal modes, as indicated by the existence of microinertia in both terms of the eigenfrequencies. This is in stark contrast to the dispersion relations of conventional elastic waves that are dominated by translation motion. In addition, we see that the chiral parameters ${B}_{2}$ and ${B}_{3}$ induce the splitting of the transverse modes.

By fitting the analytical dispersion relations and the constitutive relations with the numerical results of band structure and eigenmodes (see Supplementary Information), we retrieved the effective constitutive tensors ${B}_{{ijkl}}$,$\,{C}_{{ijkl}}$, and$\,{D}_{{ijkl}}$. We then apply these tensors to analytically evaluate the dispersion relations and the results are plotted as the solid red lines in Fig. 3c. In addition, we numerically simulated the band structures of the micropolar effective medium and results are shown as the green markers in Fig. 3c. All results agree excellently for $k \, < \, 0.15\pi /a$, demonstrating the validity of the effective medium description based on micropolar elasticity.

Under the effective medium description, the transverse modes are circularly polarized plane waves propagating in a homogeneous micropolar medium with acoustic activity. They carry well-defined spin and allows the possibility of achieving SOIs. In what follows, we demonstrate two SOI phenomena via numerical simulations and experiments. Effective medium theory based on micropolar elasticity is also applied to understand the results.

### SOI in momentum space

The transverse sound near the $\Gamma$ point in Fig. 3c can be described by an effective Hamiltonian $H=-v{{{{{\bf{S}}}}}}\cdot {{{{{\bf{k}}}}}}$ with **S** being the spin-1 operator defined as ${({S}_{i})}_{{jk}}=-{{{{{\rm{i}}}}}}{\epsilon }_{{jki}}$. The Hamiltonian indicates a coupling between the spin and linear crystal momentum **k**, which induces splitting of the eigenfrequencies $\triangle \omega \propto k$ and leads to a “negative band” for the LCP sound with spin $s=\langle {{{{{\rm{LCP}}}}}}|{{{{{\bf{S}}}}}}\cdot \hat{{{{{{\bf{k}}}}}}}|{{{{{\rm{LCP}}}}}}\rangle =+1$, as shown in Fig. 3c. Near the $\Gamma$ point, the group velocity and phase velocity take opposite signs, indicating negative refraction for a sound wave passing the metamaterial-air interface. We can define an effective refractive index $n={-v}_{0}k/{\omega }_{T}^{-}$ with ${v}_{0}$ being the speed of sound in air^28^. This acoustic activity-induced negative index is different from those derived from overlapped monopolar and dipolar resonances^43,44^ or from multipole scattering^45^. It was proposed and verified in optics^28,29^, but has been long considered impossible for sound, as longitudinal waves cannot distinguish material chirality. Next, we numerically and experimentally demonstrate negative refraction in the 3D micropolar metamaterial.

**Fig. 4:**
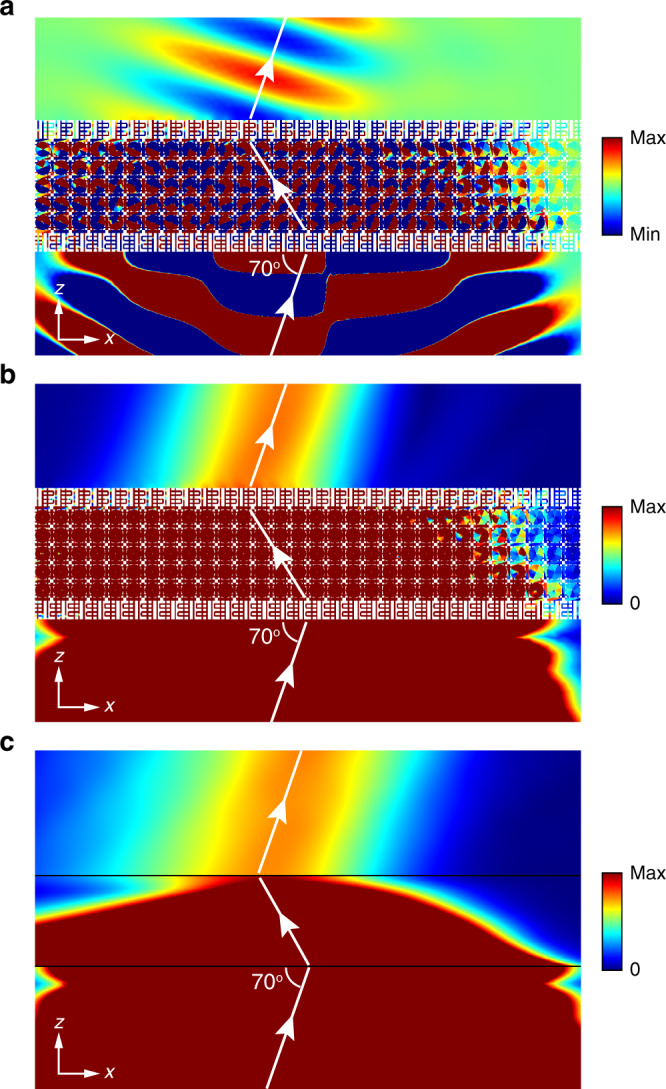
Negative refraction induced by SOI in momentum space. The real part (**a**) and the amplitude (**b**) of the pressure field for a Gaussian beam that propagates through the acoustic metamaterial with an incident angle of 70°. **c** The amplitude of pressure field in the corresponding micropolar effective medium system.

In the numerical simulation, we consider the metamaterial consisting of 5 unit cells along *z* direction and 30 unit cells along *x* direction, as shown in Fig. 4a. A periodic boundary condition is applied in the *y* direction. A Gaussian beam obliquely incidents on the metamaterial at 70°. As the sound beam is longitudinal in the air but transverse in the metamaterial, impedance mismatch happens at the interfaces. For the efficient excitation of transverse sound, we engineered the surface impedance by adding acoustic tubes (see Supplementary Information). This also guarantees that only the $s=+1$ sound is excited in the metamaterial. Figure 4a, b show the real part and the amplitude of the pressure field, respectively. The negative refraction is clearly observed. To verify the effective medium description of this phenomenon, we apply the effective parameters (same as those in Fig. 3c) to simulate the propagation of the same Gaussian beam in the micropolar effective medium. Negative refraction is seen again, as shown in Fig. 4c.

**Fig. 5:**
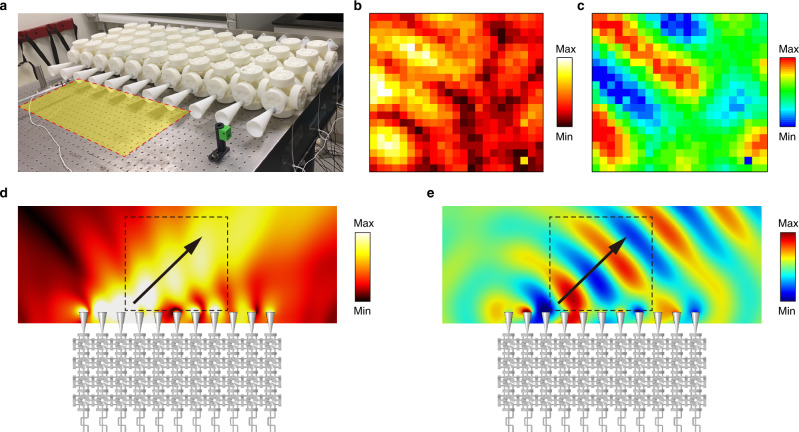
Experimental demonstration of the negative refraction. **a** A photograph of the metamaterial lattice and the measurement area (yellow colored). **b** The amplitude and (**c**) the real part of the measured pressure field. **d** The amplitude and (**e**) the real part of the simulated pressure field. The dashed boxes indicate the corresponding measurement area in the experiment.

Experimentally, we fabricated a smaller sample consisting of $11\times 4$ unit cells, as shown in Fig. 5a. This one-layer metamaterial can also induce negative refraction, as expected from the band structure of the 1D lattice system in Fig. 2d. We indeed observed the phenomenon by measuring the transmitted pressure field in the yellow zone of Fig. 5a. Figure 5b, c, respectively, show the amplitude and the real part of the pressure field. The beam with an incident angle of 40° is generated by an array of speakers. The simulation results are shown in Fig. 5d, e, where the region of experimental measurement is marked by the rectangle. Good agreement between the simulation and experimental results is seen, which confirms the negative refraction phenomenon induced by SOI.

### SOI in real space

The SOIs of transverse waves can also happen in real space. One intriguing phenomenon induced by such SOIs is the spin-dependent vortex generation in the scattering of subwavelength particles, which leads to the conversion of spin to OAM with important applications in optics such as optical manipulations and imaging^2,46–48^. It is commonly believed that airborne sound does not have this remarkable property. Here we demonstrate the real-space SOI for the transverse sound in the micropolar metamaterial.

**Fig. 6:**
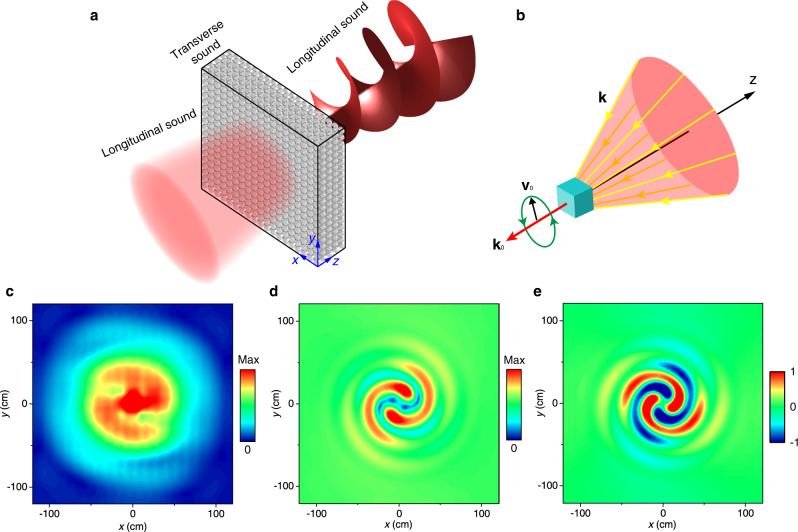
Spin-dependent vortex generation enabled by SOI in real space. **a** The schematic of the scattering system. One unit cell is removed from the center of the metamaterial to create a dipole scatterer. A Gaussian beam is normally incident on the metamaterial. **b** The schematic of the scattering of transverse sound inside the metamaterial. The blue cube denotes the scatterer. **c** The amplitude of the transmitted pressure field. **d** The velocity amplitude in the micropolar effective medium due to the interference of $s=-1$ scattered field with the background field. **e** The real part of the $s=-1$ scattered velocity field in the micropolar effective medium.

We consider the micropolar metamaterial consisting of $19\times 19\times 4$ unit cells under the normal incidence of a Gaussian beam at *f* = 655 Hz (corresponding to the frequency of the “negative band”), as shown in Fig. 6a. We remove one unit cell from the center of the metamaterial to create a subwavelength defect, as shown by the blue cube in Fig. 6b. This defect then serves as an acoustic dipole particle. Figure 6c shows the amplitude of the transmitted pressure field obtained by simulations. We notice a spiral pattern with two arms, which is a signature of an optical vortex with topological charge $q=+2.$ This phenomenon can be understood as a result of SOI mediated by the dipole particle. The longitudinal sound in air excites the transverse sound in the metamaterial that carries spin $s=+1$. The transverse sound has a velocity field ${{{{{{\bf{v}}}}}}}_{0}\,$ and a negative wave vector ${{{{{{\bf{k}}}}}}}_{0}$. It is scattered by the dipole particle, which generates scattered fields ${{{{{{\bf{v}}}}}}}_{{{{{{\rm{s}}}}}}}$ with a negative wave vector ${{{{{\bf{k}}}}}}$, as shown in Fig. 6b. The scattered field can be considered a spherical projection of the incident field: ${{{{{{\bf{v}}}}}}}_{{{{{{\rm{s}}}}}}}{{{{\propto }}}}-\hat{{{{{{\bf{r}}}}}}}\times (\hat{{{{{{\bf{r}}}}}}}\times {{{{{{\bf{v}}}}}}}_{0})$, where $\hat{{{{{{\bf{r}}}}}}}$ is the unit radial vector. The projection induces noncommutative SO(3) rotations of the incident field and leads to geometric phases that account for the spin-to-OAM conversion^5^. This process can be expressed as $|s\rangle \to {c}_{1}|s\rangle +{c}_{2}{e}^{2{{{{{\rm{i}}}}}}s\varphi }|-\!s\rangle$, where $\varphi$ is the azimuthal angle, ${c}_{1}$ and ${c}_{2}$ are the coefficients characterizing the efficiency of the SOI^49^. The second term indicates the flip of spin and the emergence of an optical vortex with topological charge $q=2s$. At the output interface, the background Gaussian beam and the scattered field are both converted to longitudinal sound, and their interference gives rise to the spiral pattern of pressure amplitude shown in Fig. 6c. To verify the results, we simulate the phenomenon in the micropolar effective medium using the same effective parameters as in Fig. 4. Similar interference pattern of the velocity field is obtained inside the micropolar effective medium, as shown in Fig. 6d. Figure 6e shows the real part of the scattered velocity field with $s=-1$, which clearly shows a $4\pi$ phase variation in the azimuthal direction and confirms the optical vortex with charge $q=+2$.

## Discussion

We have demonstrated a mechanism that transforms airborne sound into a transverse wave with rich phenomena of SOIs. The SOIs are in contrast to the pseudo-SOIs in acoustic topological insulators where hybridization of modes are employed to construct “pseudo-spins”^50^. Our idea relies on engineering acoustic resonances at the subwavelength level to emulate shear responses, thereby giving rise to a fully vectorial transverse sound that carries a spin. From a microscopic perspective, this mechanism is similar to the emergence of induced dipole moments in a dielectric medium. Notably, dipole responses have been widely leveraged for anomalous effective mass density^51^. However, those dipole moments are parallel to the propagation direction, whereas in our micropolar metamaterial, the dipoles undergo microrotation in the plane orthogonal to the propagation direction. Consequently, a total of six degrees of freedom are needed to fully characterize the transverse sound in 3D, thereby bringing richer functionalities for sound manipulations. We note that the acoustic resonators in the metamaterial unit cell also support higher-order modes (e.g., quadrupole), which can endow sound with similar transverse properties. However, these modes exist at higher frequencies where an effective medium description may encounter difficulties and diffraction effects at the interface can affect the SOI phenomena.

We anticipate more explorations of the intriguing properties of the spin-1 transverse sound. For example, an interface formed by two micropolar metamaterials can support surface acoustic waves, which may have a topological origin and interesting non-Hermitian properties under a Weyl-type representation similar to electromagnetic surface waves^52,53^. In particular, the presence of micropolar material parameters can significantly enrich the properties of the surface “acoustic plasmons”^54^. In addition, the reflection/refraction of the transverse sound at an interface can give rise to an acoustic spin-Hall effect. The canonical momentum and spin densities of the transverse sound can induce radiation forces and torques on small particles in configurations similar to the one shown in Fig. 6a^55,56^, which could give rise to counterintuitive mechanical effects that can be experimentally probed using interference methods^57,58^. The acoustic activity can enable chiral sound–matter interactions with many applications, such as chiral discrimination and sensing, acoustic manipulations of chiral particles, and acoustic circular dichroism, etc. The spin-1 sound demonstrated here can also realize the bosonic analog of Kramers doublet. We thus expect a variety of applications and extensions of the results in spin–orbit acoustics, topological acoustics, and acoustic metamaterials.

## Methods

### Micropolar effective medium theory

Near the $\Gamma (k=0)$ point, the acoustic metamaterial is approximately equivalent to a homogeneous and isotropic micropolar medium. Each point of the medium is characterized by a displacement field **u** and a microrotation field ${{{{{\mathbf{\phi }}}}}}$. Using Einstein summation convention, the strain tensor and curvature tensor can be expressed as^23^

1 $${\varepsilon }_{{ij}}=\frac{\partial {u}_{j}}{\partial {x}_{i}}-{\epsilon }_{{ijk}}{\phi }_{k},$$

2 $${\kappa }_{{ij}}=\frac{\partial {\phi }_{j}}{\partial {x}_{i}}.$$

The constitutive relations are^26,35^

3 $${\sigma }_{{ij}}={C}_{{ijkl}}{\varepsilon }_{{kl}}+{B}_{{ijkl}}{\kappa }_{{kl}},$$

4 $${m}_{{ij}}={B}_{{klij}}{\varepsilon }_{{kl}}+{D}_{{ijkl}}{\kappa }_{{kl}},$$

where the elastic constitutive tensors can be expressed as

$${C}_{{ijkl}}={C}_{1}{\delta }_{{ij}}{\delta }_{{kl}}+{C}_{2}{\delta }_{{ik}}{\delta }_{{jl}}+{C}_{3}{\delta }_{{il}}{\delta }_{{jk}},$$

5 $${B}_{{ijkl}}={B}_{1}{\delta }_{{ij}}{\delta }_{{kl}}+{B}_{2}{\delta }_{{ik}}{\delta }_{{jl}}+{B}_{3}{\delta }_{{il}}{\delta }_{{jk}},$$

$${D}_{{ijkl}}={D}_{1}{\delta }_{{ij}}{\delta }_{kl}+{D}_{2}{\delta }_{{ik}}{\delta }_{{jl}}+{D}_{3}{\delta }_{{il}}{\delta }_{{jk}}.$$

In terms of conventional notation, we have

$${C}_{1}=\lambda ,{C}_{2}=\mu +\kappa ,{C}_{3}=\mu -\kappa ,$$

6 $${B}_{1}=\eta ,{B}_{2}=\zeta +\xi ,{B}_{3}=\zeta -\xi ,$$

$${D}_{1}=\alpha ,{D}_{2}=\beta +\gamma ,{D}_{3}=\beta -\gamma .$$

Here, $\lambda$ and $\mu$ are the Lame constants; $\kappa ,{{{{{\rm{\alpha }}}}}},{{{{{\rm{\beta }}}}}}$, and $\gamma$ are the micropolar elastic constants; and $\eta ,\zeta$, and $\xi$ are the elastic constants due to material chirality. The equations governing the propagation of the sound wave in the chiral micropolar medium are given by the conservation of linear momentum and angular momentum:

7 $$\frac{\partial {\sigma }_{{ji}}}{\partial {x}_{j}}=\rho \frac{{\partial }^{2}{u}_{i}}{\partial {t}^{2}},$$

8 $$\frac{\partial {m}_{{ji}}}{\partial {x}_{j}}+{\epsilon }_{{ijk}}{\sigma }_{{jk}}={{{{{\mathscr{j}}}}}}\frac{{\partial }^{2}{\phi }_{i}}{\partial {t}^{2}},$$

where $\rho$ is the mass density and ${{{{{\mathscr{j}}}}}}$ is the microinertia density. Assuming time-harmonic forms of the displacement field ${u}_{i}={U}_{i}{e}^{{{{{{\rm{i}}}}}}{k}_{i}{x}_{i}-{{{{{\rm{i}}}}}}\omega t}$ and microrotation field ${\phi }_{i}={\Phi }_{i}{e}^{{{{{{\rm{i}}}}}}{k}_{i}{x}_{i}-{{{{{\rm{i}}}}}}\omega t}$, and using the constitutive relations, the above governing equations can be reduced to

9 $$-{k}_{j}{k}_{k}{C}_{{jikl}}{U}_{l}+({{{{{\rm{i}}}}}}{k}_{j}{\epsilon }_{{nkl}}{C}_{{jikn}}-{k}_{j}{k}_{k}{B}_{{jikl}}){\Phi }_{l}=-\rho {\omega }^{2}{U}_{i},$$

$$(i{k}_{n}{\epsilon }_{{ijk}}{C}_{{jknl}}-{k}_{j}{k}_{k}{B}_{{klji}}){U}_{l}+{{{{{\rm{i}}}}}}{k}_{j}({\epsilon }_{{nkl}}{B}_{{knji}}+{\epsilon }_{{ink}}{B}_{{nkjl}}){\Phi }_{l}$$

10 $$-({k}_{j}{k}_{k}{D}_{{jikl}}-{\epsilon }_{{ijk}}{\epsilon }_{{nml}}{C}_{{jkmn}}){\Phi }_{l}={{{{{\mathscr{-}}}}}}{{{{{\mathscr{j}}}}}}{\omega }^{2}{\Phi }_{i}.$$

Expressing ${U}_{i}$ in terms of ${\Phi }_{i}$ by using Eq. (9) and substituting it into Eq. (10), we obtain

11 $$H{{{{{\boldsymbol{\Phi }}}}}}=\left[-v{{{{{\bf{S}}}}}}\cdot {{{{{\bf{k}}}}}}+{a}_{1}{{{{{\bf{kk}}}}}}{{{{{\boldsymbol{+}}}}}}{a}_{2}{k}^{2}+O\left({k}^{3}\right)\right]{{{{{\boldsymbol{\Phi }}}}}}=\delta \omega {{{{{\boldsymbol{\Phi }}}}}},$$

where we have expanded the equation at $k\to 0$ and $\omega -{\omega }_{0}=\delta \omega \to 0$ with ${\omega }_{0}=\sqrt{2\left({C}_{2}-{C}_{3}\right){{{\mathscr{/}}}}{{{\mathscr{j}}}}}$. Here, *H* is the effective Hamiltonian, **S** is the spin-1 matrix operator defined as ${\left({S}_{i}\right)}_{{jk}}=-{{{\rm{i}}}}{\epsilon }_{{jki}},{v}=\left({B}_{2}-{B}_{3}\right)/\sqrt{2{{{\mathscr{j}}}}\left({C}_{2}-{C}_{3}\right)},{a}_{1}=\left({D}_{1}+{D}_{3}\right)/\sqrt{8{{{\mathscr{j}}}}\left({C}_{2}-{C}_{3}\right)}{{{\boldsymbol{-}}}}\sqrt{2{{{\mathscr{j}}}}\left({C}_{2}-{C}_{3}\right)}/8\rho$, and ${a}_{2}={D}_{2}/\sqrt{8{{{\mathscr{j}}}}\left({C}_{2}-{C}_{3}\right)}+\sqrt{2{{{\mathscr{j}}}}\left({C}_{2}-{C}_{3}\right)}/8\rho$. It is noted that the leading order of the effective Hamiltonian describes the SOI. The above equation gives three eigenmodes that are dominated by the microrotation of mass points, among which two are transverse waves and one is a longitudinal wave. Their dispersion relations (retained the lowest order of $k$) are

12 $${\omega }_{T}^{\pm }={\omega }_{0}\pm {vk},\,\,{\omega }_{L}={\omega }_{0}+\tau {k}^{2},$$

where $\tau =\left({D}_{1}+{D}_{2}+{D}_{3}\right)/\sqrt{8{{{{{\mathscr{j}}}}}}\left({C}_{2}-{C}_{3}\right)}$. In the low-frequency limit, microrotation vanishes in the metamaterial due to the cut-off frequencies of the resonators. Thus, $\mu ,\kappa ,\alpha ,\beta ,\gamma ,\eta ,\zeta$, and $\xi$ all vanish, only ${C}_{1}=\lambda$ (i.e., bulk modulus) remains. In this case, the metamaterial reduces to conventional acoustic metamaterial without bianisotropy.

### Effective parameters retrieval

We retrieved the effective parameters based on the numerically computed band structures and the eigenmodes. Among the total 11 material parameters, only 9 parameters (i.e., ${B}_{2},{B}_{3},{C}_{2},{C}_{3},{D}_{1},{D}_{2},{D}_{3},\rho ,{{j}}$) contribute to the microrotation-dominated waves that are responsible for the SOI phenomena. ${B}_{1}$ and ${C}_{1}$ do not play a role in the effective properties of the metamaterial for these waves. A three-step approach is applied to retrieve the effective parameters. We first evaluated the total force and torque acting on the unit cell, and applied Newton’s second law to calculate the effective mass density $\rho$ and microinertia density ${{{{{\mathscr{j}}}}}}$. Then, we fit the analytical dispersion relations with high-order corrections to the numerically computed band structures, from which the values of $\,{C}_{2}-{C}_{3},{B}_{2},{B}_{3},{D}_{2},{D}_{1}+{D}_{3}$ can be determined. To further determine the values of ${C}_{2},{C}_{3},{D}_{1}$, and ${D}_{3}$, we employ the constitutive relations of Eqs. (3) and (4), where the strain and coupling stress can be obtained via boundary averaging of the eigenmode fields. The details about the parameter retrieval can be found in Supplementary Information. For a narrow frequency region near the $\Gamma$ point, the retrieved effective parameters are approximately constants: $\rho =0.637{{{{{\rm{kg}}}}}}/{{{{{{\rm{m}}}}}}}^{3}$, ${{j}}{{{{{\mathscr{=}}}}}}5.64\times {10}^{-4}{{{{{\rm{kg}}}}}}/{{{{{\rm{m}}}}}}$, ${B}_{2}=5.91{{{{{\rm{N}}}}}}/{{{{{\rm{m}}}}}}$, ${B}_{3}=55.0{{{{{\rm{N}}}}}}/{{{{{\rm{m}}}}}}$, ${C}_{2}=-1.68\times {10}^{4}{{{{{\rm{Pa}}}}}}$, ${C}_{3}=-2.16\times {10}^{4}{{{{{\rm{Pa}}}}}}$, ${D}_{1}=20.3{{{{{\rm{N}}}}}}$, ${D}_{2}=2.69{{{{{\rm{N}}}}}}$, and ${D}_{3}=-16.2{{{{{\rm{N}}}}}}$. These material parameters are then used in full-wave numerical simulations of the micropolar effective medium to verify the band structures and SOI phenomena of the metamaterial systems.

### Numerical simulations

Full-wave numerical simulations are performed by using the finite-element package COMSOL Multiphysics (www.comsol.com) for both the metamaterial systems and the micropolar effective medium. For the resonators in Figs. 1, 3, 4, and 6, we set the radius *R* = 5 cm and height *h* = 2 cm. The period in both 1D and 3D metamaterials is $a=12.1\,{{{{{\rm{cm}}}}}}$. The tubes have radii *r* = 0.2 cm. For the chiral resonator, the upper surface is twisted $\pi /2$ with respect to the bottom surface. The Gaussian beam in Figs. 4 and 6 has a beam width $w=1.2\lambda$. A sound-hard boundary condition is applied on all the boundaries of the resonators and tubes. Floquet periodic boundary conditions are applied to the 1D lattice and 3D metamaterials to compute the band structures. To compute the band structures of the micropolar effective medium and to simulate the associated SOIs phenomena, we developed weak-form formulations for the micropolar constitutive relations and momentum conservation equations, which are then implemented using COMSOL. The band structures of the micropolar medium are calculated by considering a unit cell made of homogenous and isotropic micropolar medium with the retrieved effective parameters.

### Experiments

The 1D lattice and 3D metamaterial were fabricated by using 3D printing. The resonators and connecting tubes are made of acrylonitrile butadiene styrene plastics, which were then assembled to form the structures in Figs. 2a and 5a. The fabricated units correspond to a scaled version of the units in Figs. 1a, c and  3a with $R=3.5\,{{{{{\rm{cm}}}}}},{h}=1.75\,{{{{{\rm{cm}}}}}},{r}=0.4\,{{{{{\rm{cm}}}}}}$, and $a=10.7\,{{{{{\rm{cm}}}}}}$. For the band structures of the 1D lattices, we excite the lattice using a loudspeaker at one end. The signal is generated by a waveform generator (Keysight 33500B) as a short pulse covering the frequency range of interest. We then measure the pressure responses with a microphone and a digital oscilloscope (Keysight DSO2024A) at all 24 unit cells with one measurement point per cell. Then, we perform a 2D Fourier transform to obtain the dispersion curves, which show the band structures (Fig. 2c, d). For the negative refraction experiment, we used an array of 11 loudspeakers to generate an obliquely incident Gaussian beam. Each speaker was driven by an independent channel of a computer sound interface (MOTU 16A). Both the amplitudes and phases of the output signal from each channel were precisely controlled by a PC (via a MATLAB program) to generate the targeted Gaussian beam with a tilted phase profile, in order to emulate oblique incidence at the chosen angle. On the far side of the metamaterial, small horns are connected to each unit cell, to improve impedance matching between the metamaterial and the air. The tabletop and a top plate (removed in Fig. 5a to show the metamaterials) form a two-dimensional waveguide in the output region for the better observation of the negative refracted field profile. A microphone is carried by a translational stage to raster-map the output beam profiles.

## Supplementary information

Supplementary Information

## Data Availability

The authors declare that all data supporting the findings of this study are available within the paper and its Supplementary Information files. Additional data related to this paper are available from the corresponding authors upon reasonable request.
