# Supplementary material for: Spin-orbit interactions of transverse sound
Source: Nat Commun. 2021 Oct 21;12:6125. doi: 10.1038/s41467-021-26375-9 (PMC8531336; doi:10.1038/s41467-021-26375-9)
Supplement: Supplementary file 1 — Supplementary Information [file 41467_2021_26375_MOESM1_ESM.pdf]

Supplementary Information for

Spin-orbit interactions of transverse sound

Shubo Wang[1,*], Guanqing Zhang[2], Xulong Wang[2], Qing Tong[1], Jensen Li[3], and Guancong Ma[2,*]

[1] Department of Physics, City University of Hong Kong, Tat Chee Avenue, Kowloon, Hong Kong, China

[2] Department of Physics, Hong Kong Baptist University, Kowloon Tong, Hong Kong, China

[3] Department of Physics, The Hong Kong University of Science and Technology, Clear Water Bay, Hong Kong, China

*Correspondence should be addressed to:
Shubo Wang (shubwang@cityu.edu.hk)
Guancong Ma (phgcma@hkbu.edu.hk)

Supplementary Note 1: Retrieval of effective material parameters

To characterize the material properties of the micropolar metamaterial, we need 9 parameters (i.e., $B_1, B_2, B_3, C_1, C_2, C_3, D_1, D_2, D_3$) to describe the elasticity and two parameters (i.e., mass density ρ and microinertia density j) for the dynamic equations. Here, we apply a three-step method to determine the values of these parameters. The retrieved parameters are then used for numerical simulations of the micropolar effective medium. A comparison between the results of the effective medium and that of the metamaterial can validate our understanding of the metamaterial's acoustic properties. Since the metamaterial is approximately isotropic near Γ point (as seen from the isofrequency contours shown in Fig. 3(d) and 3(e) of the main text), we consider the LCP sound with $s = +1$ propagating in the +*x* direction and use the eigenfields in the following evaluations to obtain the effective parameters.

The mass density ρ can be determined via Newton's 2nd law

$$\rho = \frac{m}{V} = \frac{F_x}{\ddot{u}_x a^3} = \frac{-F_x}{a^3 \omega^2 u_x}. \tag{S1}$$

Here a is the size of the unit cell, F_x is the *x* component of the net force acting on the unit cell, which can be calculated as $F_x = \hat{\mathbf{x}} \cdot \int \hat{\mathbf{n}} p dS$, $\hat{\mathbf{x}}$ is the unit vector of *x* axis, $\hat{\mathbf{n}}$ is the inward normal unit vector, p is the pressure, and the integral is evaluated over all surfaces of the unit cell. u_x is the averaged displacement of the unit cell along *x* direction that is defined as

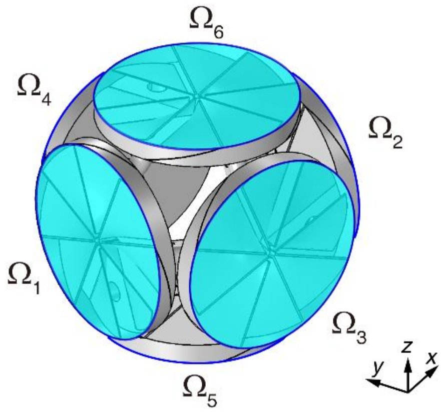

Fig. S1. Unit cell of the micropolar metamaterial.

$u_x = \frac{1}{6a^2}\hat{\mathbf{x}}\cdot\int \mathbf{u}dS$, and the integral is evaluated over all 6 surfaces of the unit cell (corresponding to the blue-colored surfaces in Fig. S1).

The micro-rotation inertia density $\jmath$ can be determined as

$$\jmath = \frac{J}{V} = \frac{1}{a^3}\sum_{i=1}^{3}\frac{T_x^i}{\ddot{\Phi}_x^i} = \frac{-1}{a^3\omega^2}\sum_{i=1}^{3}\frac{T_x^i}{\Phi_x^i}, \tag{S2}$$

where $T_x^i = \hat{\mathbf{x}}\cdot\int(\mathbf{r}-\mathbf{r}_i)\times\hat{\mathbf{n}}pdS$ is the x-component of the torque exerted on ith resonator (each unit cell has 3 resonators) with respect to its center $\mathbf{r}_i$, $\Phi_x^i = \frac{1}{V}\hat{\mathbf{x}}\cdot\int\frac{(\mathbf{r}-\mathbf{r}_i)\times\mathbf{u}}{|\mathbf{r}-\mathbf{r}_i|^2}dV$ is the average rotation of the volumetric air inside the resonator with respect to the center, and V is the volume of the resonator.

In the second step, we employ the dispersion relations to retrieve part of the constitutive tensor parameters. The dispersion relations Eq. (12) in the main text are derived by keeping the leading orders up to $\mathrm{O}(k^2)$, which already can capture the underlying physics and reproduce the key features of the band structures. To use it for parameter retrieval, we adopt the following dispersion relations with leading orders up to $\mathrm{O}(k^3)$

$$\omega_T^{\pm} = \sqrt{\frac{2(C_2-C_3)}{\jmath}} \pm \frac{B_2-B_3}{\sqrt{2\jmath(C_2-C_3)}}k + \frac{(C_2-C_3)^2\jmath - [(B_2-B_3)^2 - 2(C_2-C_3)D_2]\rho}{4\rho\sqrt{2\jmath(C_2-C_3)^3}}k^2 \pm \frac{(B_2+3B_3)(C_2-C_3)^2\jmath + (B_2-B_3)[(B_2-B_3)^2-2(C_2-C_3)D_2]\rho}{8\rho\sqrt{2\jmath(C_2-C_3)^5}}k^3, \tag{S3}$$

$$\omega_L = \sqrt{\frac{2(C_2-C_3)}{\jmath}} + \frac{D_1+D_2+D_3}{2\sqrt{2\jmath(C_2-C_3)}}k^2, \tag{S4}$$

The above equations are fitted to the numerically obtained band structures in Fig. 3(c). The fitting can give the values of $B_2, B_3, D_2, C_2 - C_3$ and $D_1 + D_3$. We note that B_1 and C_1 do not appear in the above dispersion relations, which indicates that their contribution to the considered transverse modes is negligible. This is confirmed by numerical simulations of the micropolar effective medium.

To determine the values of C_2, C_3, D_1 and D_3, we then employ the constitutive relations in Eqs. (3) and (4) of the main text. The following two equations are sufficient to determine their values:

$$m_{12} = B_2\varepsilon_{12} + B_3\varepsilon_{21} + D_2\kappa_{12} + D_3\kappa_{21}, \tag{S5}$$

$$\sigma_{12} = C_2\varepsilon_{12} + C_3\varepsilon_{21} + B_2\kappa_{12} + B_3\kappa_{21}, \tag{S6}$$

where the couple stress m_{12} is evaluated as

$$m_{12} = \frac{1}{2a^2}\hat{\mathbf{y}} \cdot \left[\int_{\Omega_2} (\mathbf{r} - \mathbf{r}_i) \times \hat{\mathbf{n}} p dS - \int_{\Omega_1} (\mathbf{r} - \mathbf{r}_i) \times \hat{\mathbf{n}} p dS\right], \tag{S7}$$

where "Ω_1" and "Ω_2" denote the surfaces of the two half resonators as labeled in Fig. S1. The force stress σ_{12} is evaluated as

$$\sigma_{12} = \frac{1}{2L}\hat{\mathbf{y}} \cdot \left[\int_{x=+\frac{a}{2}} \hat{\mathbf{n}} p dl - \int_{x=-\frac{a}{2}} \hat{\mathbf{n}} p dl\right], \tag{S8}$$

where the two integrals are evaluated over all edges of the boundary surface at $x = +a/2$ and $x = -a/2$, respectively. L is the total length of the edges of each boundary surface. The strain ε_{12} and ε_{21} are evaluated as

$$\varepsilon_{12} = \frac{1}{a^3}\hat{\mathbf{y}} \cdot \left[\int_{x=+\frac{a}{2}} \mathbf{u} dS - \int_{x=+\frac{a}{2}} \mathbf{u} dS\right], \varepsilon_{21} = \frac{1}{a^3}\hat{\mathbf{x}} \cdot \left[\int_{y=+\frac{a}{2}} \mathbf{u} dS - \int_{y=+\frac{a}{2}} \mathbf{u} dS\right] \tag{S9}$$

and the curvature κ_{12} and κ_{21} are evaluated as

$$\kappa_{12} = \frac{2}{aV}\hat{\mathbf{y}} \cdot \left[\int_{\Omega_2} \frac{(\mathbf{r} - \mathbf{r}_i) \times \mathbf{u}}{|\mathbf{r} - \mathbf{r}_i|^2} dV - \int_{\Omega_1} \frac{(\mathbf{r} - \mathbf{r}_i) \times \mathbf{u}}{|\mathbf{r} - \mathbf{r}_i|^2} dV\right], \tag{S10}$$

$$\kappa_{21} = \frac{2}{aV}\hat{\mathbf{x}} \cdot \left[\int_{\Omega_4} \frac{(\mathbf{r}-\mathbf{r}_i)\times\mathbf{u}}{|\mathbf{r}-\mathbf{r}_i|^2} dV - \int_{\Omega_3} \frac{(\mathbf{r}-\mathbf{r}_i)\times\mathbf{u}}{|\mathbf{r}-\mathbf{r}_i|^2} dV \right]. \tag{S11}$$

where "Ω_3" and "Ω_4" denote the surfaces of the two half resonators as labelled in Fig. S1. Using Eqs. (S5)-(S11), we determined the values of the remaining effective parameters C_2, C_3, D_1 and D_3.

Supplementary Note 2: Surface impedance engineering

Since the sound inside the micropolar metamaterial is a transverse wave while the sound in air is a longitudinal wave, impedance mismatch occurs at the air-metamaterial interface. Thus, it is difficult to excite the transverse wave by direct illumination of sound waves from air. The excitation efficiency can be improved by engineering the surface impedance of the metamaterial. As shown in Fig. S2, we tuned the length of the four connection tubes of the interface resonators to introduce a phase gradient of $(0, \pi/2, \pi, 3\pi/2)$ to the longitudinal input wave. This will preferably excite the LCP transverse sound that gives rise to the negative refraction and vortex generation. A similar design is applied to the output interface to improve the transmission from the metamaterial to the air. In the experiments, as shown in Fig. 5(a) and (d), we adopt a simplified design and only used two tubes of different length to couple the incident longitudinal sound into the metamaterial, and one horn-shaped tube is used at the output interface to improve the transmission of the transverse sound to air.

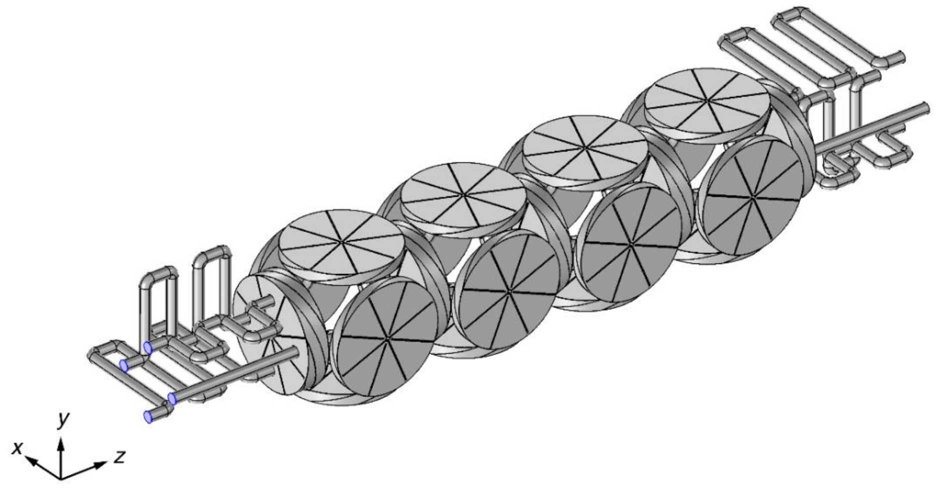

Fig. S2. Surface impedance engineering of the metamaterial.

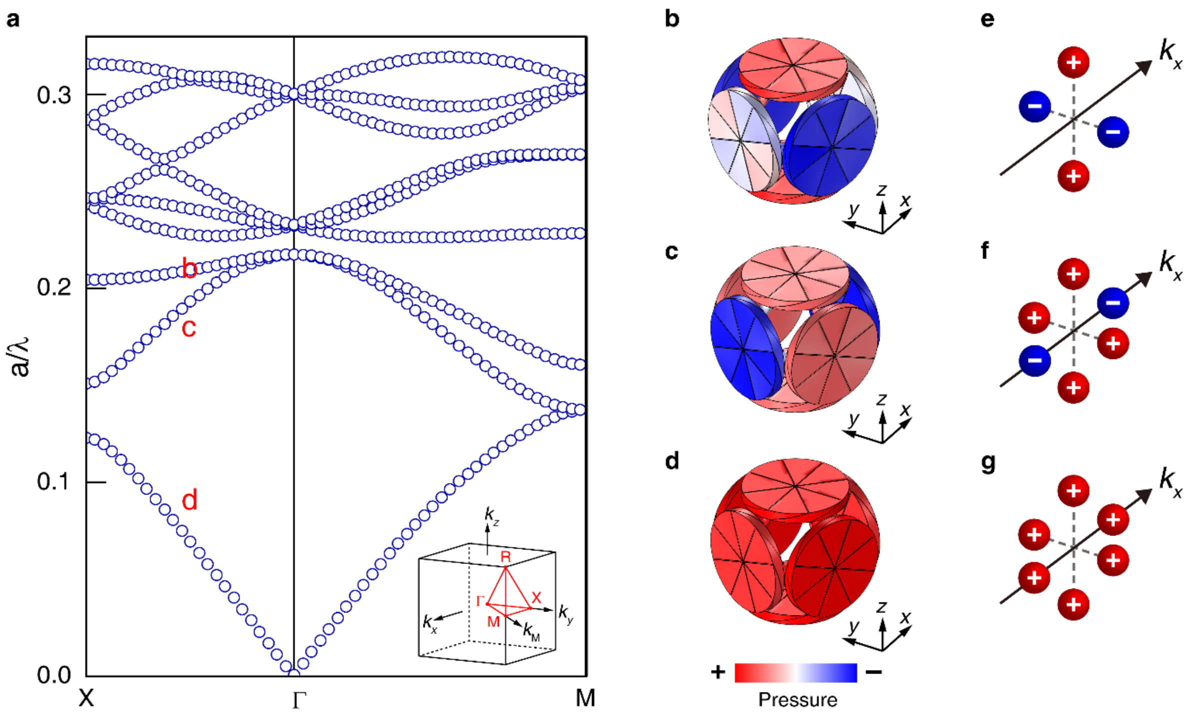

Fig. S3. Eigenmodes of the lowest three bands for the 3D metamaterials. (a) The band structure of the metamaterial (same as Fig. 3(c) in the main text). (b)(e) Eigen-ressure field and monopole distribution of the 3rd band (labelled as "b" in the band structure), corresponding to transverse compression/expansion of the unit cell. (c)(f) Eigen-pressure field and monopole distribution of the 2nd band (labelled as "c" in the band structure), corresponding to longitudinal compression/expansion of the unit cell. (d)(g) Eigen-pressure field and the monopole distribution of the 1st band (labelled as "d" in the band structure), corresponding to isotropic compression/expansion of the unit cell.
